# Genetic Interactions between Neurofibromin and Endothelin Receptor B in Mice

**DOI:** 10.1371/journal.pone.0059931

**Published:** 2013-03-28

**Authors:** Mugdha Deo, Jenny Li-Ying Huang, Catherine D. Van Raamsdonk

**Affiliations:** Department of Medical Genetics, University of British Columbia, Vancouver, Canada; Duke University Medical Center, United States of America

## Abstract

When mutations in two different genes produce the same mutant phenotype, it suggests that the encoded proteins either interact with each other, or act in parallel to fulfill a similar purpose. Haploinsufficiency of Neurofibromin and over-expression of Endothelin 3 both cause increased numbers of melanocytes to populate the dermis during mouse development, and thus we are interested in how these two signaling pathways might intersect. Neurofibromin is mutated in the human genetic disease, neurofibromatosis type 1, which is characterized by the development of Schwann cell based tumors and skin hyper-pigmentation. Neurofibromin is a GTPase activating protein, while the Endothelin 3 ligand activates Endothelin receptor B, a G protein coupled receptor. In order to study the genetic interactions between endothelin and neurofibromin, we defined the deletion breakpoints of the classical *Ednrb piebald lethal* allele (*Ednrb^s-l^*) and crossed these mice to mice with a loss-of-function mutation in neurofibromin, *Dark skin 9* (*Dsk9*). We found that Neurofibromin haploinsufficiency requires Endothelin receptor B to darken the tail dermis. In contrast, Neurofibromin haploinsufficiency increases the area of the coat that is pigmented in Endothelin receptor B null mice. We also found an oncogenic mutation in the G protein alpha subunit, GNAQ, which couples to Endothelin receptor B, in a uveal melanoma from a patient with neurofibromatosis type 1. Thus, this data suggests that there is a complex relationship between Neurofibromin and Endothelin receptor B.

## Introduction

Pigment cells arise during mammalian development through one of two known lineages. One lineage arises directly from multipotent neural crest cells, while the other lineage initiates within bipotential melanoblast-Schwann cell precursors [1], [2]. Pigment cells initially migrate through the dermis, and then later enter the epidermis and hair follicles, which are epidermal appendages [2], [3], [4], [5]. Mature pigment cells, called melanocytes, produce melanin. Melanocytes in the hair follicles pigment the hair, while melanocytes in the dermis and the inter-follicular epidermis determine skin color.

The Endothelin receptor B (*Ednrb*) is a seven transmembrane G protein coupled receptor expressed in melanocytes [6]. Ednrb is activated by the Endothelin 3 (*Edn3*) ligand [7]. Endothelin signaling transmits survival/proliferation signals into melanocytes by way of heterotrimeric G proteins, including the alpha subunits, Gnaq and Gna11 [8], [9]. Mice lacking either *Edn3* or *Ednrb* have a very similar phenotype and are hypo-pigmented due to a lack of melanocytes during development [10], [11], [12]. The coats of mice completely lacking *Ednrb* (the *Ednrb^s-l^* allele) range from being completely white to being white with small pigmented patches on the head and/or rump [12]. In addition, these mice exhibit a completely unpigmented glabrous skin (tails, ears, and feet) [13].

Several lines of evidence suggest that endothelin signaling plays an important role in melanocyte survival in the dermis, but has little, if any, effect on melanocytes located in the inter-follicular epidermis. First, transgenic over-expression of *Edn3* in keratinocytes causes hyper-pigmentation of the dermis, but not of the inter-follicular epidermis [14]. Second, there are no white coat patches when *Ednrb* knockout occurs after E12.5, when melanoblasts, immature melanocytes, first migrate into the epidermis [15]. And finally, activating mutations in *Gnaq* and *Gna11* cause dermal, but not epidermal, hyper-pigmentation [13].The dermal hyper-pigmentation of *Edn3* over-expressing mice dissipates when the transgene is shut off, indicating that endothelin signaling continues to regulate the density of melanocytes in the adult dermis [14].

Neurofibromin (*Nf1*) is a GTPase activating protein that also regulates pigmentation [16], [17], [18]. In humans, inherited heterozygous mutations in *NF1* cause neurofibromatosis type 1, which is characterized by skin hyper-pigmentation and Schwann cell based tumors. These phenotypes are caused by a combination of haploinsufficiency and somatic loss of heterozygosity [19], [20]. *Nf1* mutant mice exhibit a darker dermis and epidermis [21]. Conditional haploinsufficiency of *Nf1* induced in Schwann cell-melanoblast precursors around E11.5 recapitulates the dark dermis phenotype of *Nf1*+/− mice, while conditional *Nf1* haploinsufficiency induced in already committed melanocytes does not [21]. This indicates that *Nf1* haploinsufficiency has its effect on dermal pigmentation during a limited period of time. A developmental connection between Schwann cells and melanocytes might underlie the susceptibility of these two types of cells to *Nf1* loss. However, the cell type(s) required to recapitulate the darker epidermis phenotype have yet to be identified.

Because haploinsufficiency of neurofibromin and over-expression of Endothelin 3 both cause increased numbers of melanocytes to populate the dermis during mouse development, we are interested in how these two signaling pathways might intersect. To address this, we have examined genetic interactions between *Nf1* and *Ednrb* in mice.

## Results

### Determination of the *Ednrb^s-l^* Deletion Breakpoints

According to southern blot analysis, the entire coding region of the *Ednrb* gene is deleted in the *Ednrb^s-l^* (*piebald lethal*) allele, which is commercially available [12], [22]. To be able to use this allele in our crosses, we needed to determine the location of the *Ednrb^s-l^* deletion breakpoints, in order to verify that no other genes are included in the deletion and to permit genotyping by PCR (polymerase chain reaction).

Our strategy was to determine where PCR either did, or did not, produce a product in DNA from *Ednrb^s-l^/Ednrb^s-l^* mice, using DNA from wildtype mice as a positive control. We designed our PCR primer pairs using sequence from the Ensembl mouse genome database (m37). Beginning 20 Mb away from *Ednrb* on either side, we sequentially designed primer pairs at denser intervals, targeting the region between the last known primer pair that produced a product and first known primer pair that failed. After several rounds, we were able to narrow this deletion flanking region to ∼1 kb on either side of the deletion. Next, we designed several primer pairs in which the left primer was positioned in one deletion flanking region and the right primer was positioned in the other. Some of these primer pairs produced a product in *Ednrb^s-l^/Ednrb^s-l^* DNA, but not wildtype DNA. We sequenced these PCR products, and found that they contained the breakpoints.

**Figure 1:**
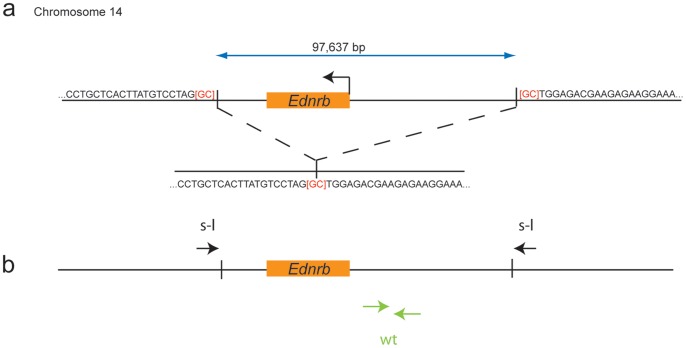
Molecular characterization of the *Ednrb^s-l^* allele. **a)** The *Ednrb^s-l^* deletion event consisted of the removal of 97,637 base pairs in total, beginning 15.72 kb upstream of *Ednrb* and continuing 54.98 kb downstream. The bases immediately surrounding the removed sequence are given. It is not possible to tell which flanking [GC] was removed during the deletion, which is indicated with brackets. **b)** The location of primer pairs used to genotype the *Ednrb^s-l^* allele versus the wildtype *Ednrb* allele is shown. Two separate PCR reactions are used to genotype each sample: one that amplifies only the wildtype allele (green, wt) and one that amplifies only the *Ednrb^s-l^* allele (black, s-l).

The *Ednrb^s-l^* deletion encompasses 97,637 base pairs of chromosome 14 (Figure 1a). According to the Ensembl genome database (m37), no other genes are located in the region that is deleted. Subsequently, for each animal to be genotyped, we used two different PCR reactions. One contains primers that span the deletion and is positive only if the *Ednrb^s-l^* allele is present, while the other contains primers within the deleted region and is positive only if the wildtype allele is present (Figure 1b).

### Interactions between *Ednrb^s-l^* and *Nf1^Dsk9^*

To study genetic interactions between *Nf1* and *Ednrb*, we made use of a loss-of-function *Nf1* allele, *Nf1^Dsk9^*. The *Dsk9* mutation is a missense in the GTPase accelerating protein related domain (GRD) of neurofibromin [21]. We crossed *Nf1^Dsk9^*/+ mice to *Ednrb^s-l^*/+ and then crossed the resulting *Ednrb^s-l^*/+; *Nf1^Dsk9^*/+ mice to *Ednrb^s-l^*/+ mice. The coats of *Ednrb^s-l^*/*Ednrb^s-l^* mice range from being completely white to being white with small pigmented patches on the head and/or rump [12], [22].

**Figure 2:**
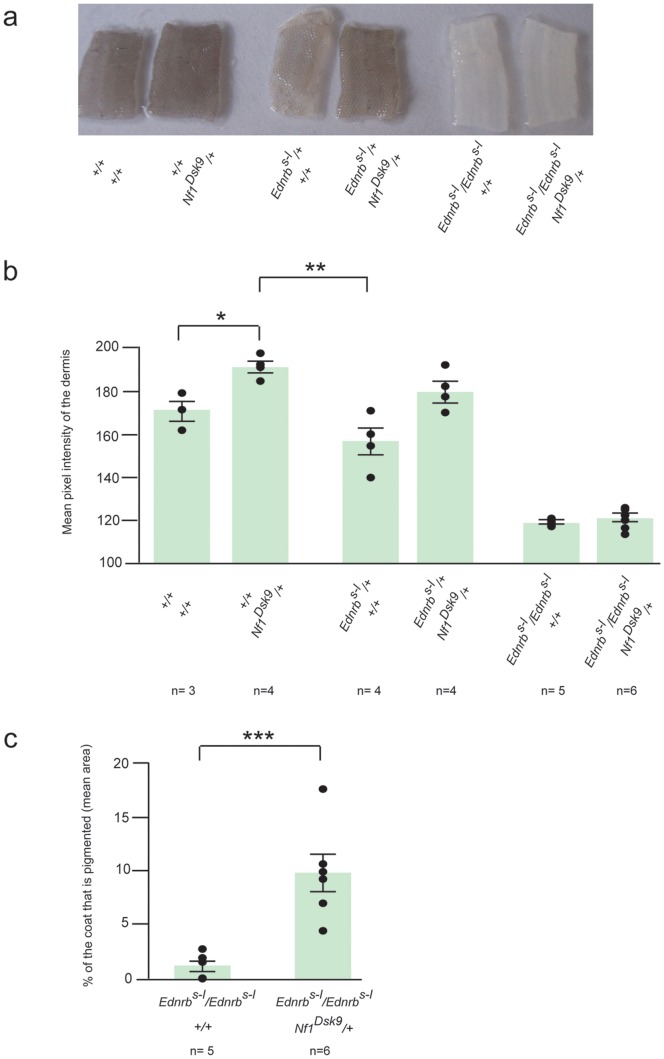
Genetic interactions between *Ednrb^s-l^* and *Nf1^Dsk9^*. **a)** Representative tail dermis of 2–3 week old mice of the indicated genotypes is shown. No pigmentation is observed in *Ednrb^s-l^/Ednrb^s-l^* tail dermis, with or without *Nf1^Dsk9^*. **b)** The mean pixel intensity of tail dermis (+/− S.E.M.) of 2–3 week old mice is shown. The mean pixel intensity of +/+; *Nf1^Dsk9^*/+ dermis is greater than +/+; +/+ dermis (p = 0.0328*, student’s ttest) and *Ednrb^s-l^*/+; +/+ dermis (p = 0.00748**, student’s ttest), while the mean pixel intensity of *Ednrb^s-l^*/+; *Nf1^Dsk9^*/+ dermis is not significantly different than +/+; +/+. The pixel intensity is not zero in *Ednrb^s-l^/Ednrb^s-l^* tail dermis, with or without *Nf1^Dsk9^*, because the photograph is not completely white in terms of pixels, even while there is no melanin. **c)** The percent of the coat that is pigmented (mean area +/− S.E.M.) in *Ednrb^s-l^/Ednrb^s-l^*; +/+ and *Ednrb^s-l^/Ednrb^s-l^*; *Nf1^Dsk9^*/+ mice is shown. On average, spots of coat pigmentation account for 9.8% of the area of the coat in *Ednrb^s-l^*/*Ednrb^s-l^*; *Nf1^Dsk9^/*+ mice, compared to 1.1% in *Ednrb^s-l^*/*Ednrb^s-l^*; *+/*+ mice (p = 0.003925***, student’s ttest). No spotting was observed in any other genotype (data not shown). In b and c, individual dots in graphs represent different animals, the total number of which is given by n for each genotype.

In the progeny from this cross, we measured the mean pixel intensity of group photographed pieces of tail dermis, split from the epidermis using sodium bromide. The mean pixel intensity correlates with the skin color of the sample (Figure 2a versus b) [13], [21] and permits statistical analysis. The mean pixel intensity of +/+; *Nf1^Dsk9^*/+ dermis is greater than +/+; +/+ dermis and *Ednrb^s-l^*/+; +/+ dermis (Figure 2a–b, p = 0.0328 and p = 0.00748, respectively, student’s ttest), while the mean pixel intensity of *Ednrb^s-l^*/+; *Nf1^Dsk9^*/+ dermis is not significantly different than +/+; +/+. This suggests an additive effect of *Ednrb^s-l^*/+ with *Nf1^Dsk9^*/+. As expected, the tail dermis of *Ednrb^s-l^*/*Ednrb^s-l^* mice is completely unpigmented (Figure 2a) [13]. The tail dermis of *Ednrb^s-l^*/*Ednrb^s-l^*; *Nf1^Dsk9^/*+ mice also lacks visible pigment (Figure 2a). Therefore, *Nf1^Dsk9^*/+ is not able to compensate for a complete lack of *Ednrb* in the tail dermis.

In addition, we observed that, on average, spots of coat pigmentation account for 9.8% of the total area of the coat in *Ednrb^s-l^*/*Ednrb^s-l^*; *Nf1^Dsk9^/*+ mice, as compared to 1.1% in *Ednrb^s-l^*/*Ednrb^s-l^*; *+/*+ mice (Figure 2c, p = 0.003925, student’s ttest). Spots of coat pigmentation in both *Ednrb^s-l^*/*Ednrb^s-l^*; *Nf1^Dsk9^/*+ and *Ednrb^s-l^*/*Ednrb^s-l^*; *+/*+ mice were restricted to the head and rump regions [12], [22].

### *GNAQ* Mutation in NF1 Uveal Melanoma

There is an interesting association between dermal hyper-pigmentation and uveal melanoma, which is a melanoma of the uveal tract of the eye. For example, the nevus of Ota, a hyperplasia of the dermis, is associated with an increased risk of uveal melanoma in people of Western European descent [23]. In addition, constitutively active, oncogenic mutations in either *GNAQ* or *GNA11* are found in ∼75% of common nevi of the dermis and in ∼83% of uveal melanomas [24]. Furthermore, constitutively active mutations in GNAQ/GNA11 [24], [25] and loss of function mutations in NF1 [26] both lead to hyper-active MAP kinase signaling. To examine whether oncogenic *GNAQ* or *GNA11* mutations are required in uveal melanomas bearing *NF1* mutations, we examined a uveal melanoma tumor from a patient with neurofibromatosis type 1, which is known to be caused by inherited heterozygous mutations in *NF1* [19], [20]. There are multiple reports of uveal melanoma in patients with neurofibromatosis type 1 in the literature, despite the rarity of uveal melanoma. For examples, see [27], [28], [29].

**Figure 3:**
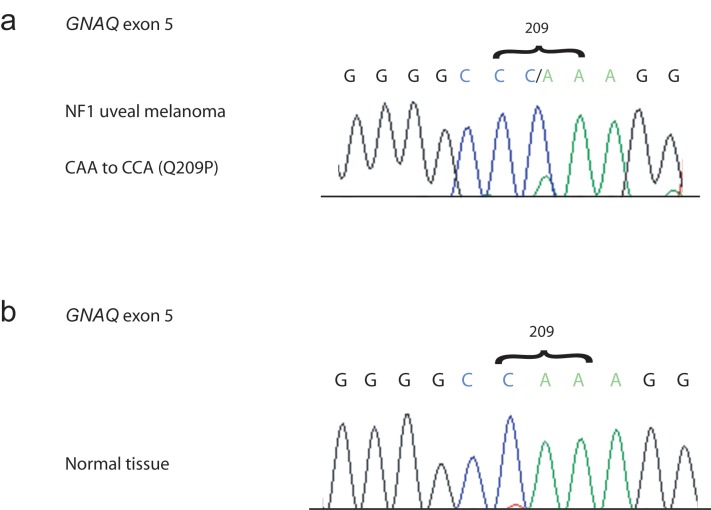
Analysis of *GNAQ* and *GNA11* in a *NF1* uveal melanoma. **a)** DNA was extracted from a uveal melanoma from a patient with neurofibromatosis type 1. The DNA was used to sequence exons 4 and 5 of *GNAQ* and *GNA11*. The tumor exhibited a CAA to CCA (glutamine to proline) substitution in *GNAQ* exon 5 at residue 209, a known oncogenic hotspot. No other mutations in *GNAQ* or *GNA11* were found. **b)** DNA extracted from surrounding normal tissue of the patient exhibited the expected sequence at *GNAQ* 209 (CAA), indicating that the Q209P mutation in the tumor is somatic.

The uveal melanoma from a patient with neurofibromatosis type 1 that we studied was previously described [30] and was available in the pathology archives at the Vancouver General Hospital. We sequenced *GNAQ* and *GNA11* in DNA extracted from the formalin-fixed, paraffin-embedded sample. We found that this tumor bears a somatic glutamine 209 to proline substitution in GNAQ (GNAQ^Q209P^), which is known to cause constitutive activity [25], [31], [32] (Figure 3a–b). This finding suggests that even in the context of *NF1* haploinsufficiency, constitutive activity of GNAQ plays an important oncogenic role in uveal melanoma.

## Discussion

We have studied the genetic interactions between *Ednrb* and *Nf1* in pigmentation, examining the result of bringing loss of function mutations in *Ednrb* and *Nf1* together in mice. When mutant alleles of two different genes are brought together in an animal, one possible outcome is that the phenotype of one allele is expressed while the other is not. This is referred to as epistasis. In our experiments, we found that *Nf1^Dsk9^* haploinsufficiency requires *Ednrb* to darken the tail dermis of mice (Figure 2a,b). Thus, *Ednrb* is epistatic to *Nf1* with respect to dermis skin color. Since the dermis of *Ednrb^s-l^/Ednrb^s-l^* mice is completely albino, this suggests that endothelin signaling is required prior to or in parallel with *Nf1* to support melanoblast formation/survival/proliferation in the dermis during development. However, we also observed that *Nf1* haploinsufficiency increases the amount of pigmented coat spots in mice that lack *Ednrb* (Figure 2c). Since *Ednrb* is not absolutely required for coat pigmentation, *Nf1^Dsk9^* appears to be able to increase melanocyte occupancy of hair follicles during development, in the absence of *Ednrb*. Thus, our data suggests that these two pathways do not act in concert all the time. This is supported by the finding of a constitutively active GNAQ mutation in an *NF1*+/− uveal melanoma, which otherwise might be redundant, if EDNRB/GNAQ signaling completely overlapped with NF1 [8], [9].

How might *Ednrb* be essential for dermal pigmentation in the tail, but not hair follicle occupancy? It is likely caused by the reduced requirement for *Ednrb* in the head and rump regions, as compared with the rest of the body. During embryogenesis, *Ednrb^s-l^/Ednrb^s-l^* embryos exhibit greatly reduced numbers of melanoblasts, at or before embryonic day (E) 10.5 [10]. This suggests that there is an early requirement for *Ednrb* in melanocyte development. However, melanoblasts expressing the melanocyte marker, dopachrome tautomerase (Dct), persist in the head and rump skin of *Ednrb^s-l^/Ednrb^s-l^* embryos [10].

This varied regional requirement for *Ednrb* could be due to environmental differences, and/or to differences in the origin of the melanoblasts themselves. The trunk dermis is derived from the somatic dermamyotome, while the head dermis is derived from the cranial neural crest. At least some of the melanoblasts in the head arise from Schwann cell precursors associated with cranial nerves, while others appear to arise directly from neural crest cells, such as those at the midbrain-hindbrain border [33]. Newly formed melanoblasts located close to cranial nerves exhibit a greater reduction in number in *Ednrb^tm1Nrd^/Ednrb^tm1Nrd^* embryos compared to the other melanoblasts in the head, suggesting that the non-Schwann cell derived melanocytes have a reduced requirement for Ednrb [33].

The colonization of hair follicles by melanoblasts occurs only when the hair follicles are first forming [34]. The percent of the coat that is pigmented reflects the number of melanoblasts that were available in the epidermis at that stage. Each self-contained hair follicle retains its occupied or unoccupied status into adulthood. Endothelin signaling appears to be dispensable for melanocytes once they are located in the epidermis [14], [15]. Thus, melanoblasts in the head and rump regions that manage to develop/survive in the absence of endothelin signaling should be able to expand freely to their maximum potential once they enter the epidermis [2], [3], [4], [5].

We have found that *Nf1* haploinsufficiency increases the area of the head and rump coat that is pigmented in *Ednrb* null mice. This indicates that melanoblasts with a reduced requirement for Ednrb can be stimulated by *Nf1* haploinsufficiency to increase in number in the epidermis. Given the findings of others [33], described above, this suggests there might be a role for *Nf1* outside Schwann cell derived melanoblasts. Because Schwann cell precursors arise from neural crest cells, there is currently no cre line that would specifically target melanoblasts arising directly from the neural crest, which could be used to test this hypothesis.

In summary, our data suggests that there is a complex relationship between Nf1 and endothelin signaling, and that there are multiple roles for *Nf1* in pigmentation. If one copy of *Nf1* is lost, more melanoblasts are produced from Schwann cell precursors, which darkens the dermis [21]. However, these melanoblasts require Ednrb for their production and/or their survival/proliferation. If both copies of *Nf1* are lost, on-going melanocyte proliferation/survival is enhanced in both the dermis and the epidermis of the tail, via a melanocyte cell autonomous mechanism [21]. Finally, on a background with reduced melanoblast numbers, *Nf1* haploinsufficiency can increase the number of melanoblasts in the head and rump epidermis, via an *Ednrb*-independent mechanism.

## Materials and Methods

### Mouse Husbandry

This study was carried out in strict accordance with the recommendations of the Canadian Animal Care Committee. All experiments were preformed under the approval of the CACC at the University of British Columbia (Protocol A09-0893) in a barrier facility, with environmental enrichment. In all experiments, we collected samples between 2 and 3 weeks of age, before the *Ednrb^s-l^*/*Ednrb^s-l^* mice develop megacolon. *Ednrb^s-l^* mice were obtained from the Jackson Laboratories [22]. *Nf1^Dsk9^* mice were recovered during an ENU mutagenesis screen at the Institute for Experimental Genetics in Germany [35]. Both strains were made predominantly C57Bl/6J.

### Genotyping

Genomic DNA from ear skin biopsies or embryonic membranes was extracted using Qiagen DNeasy Blood and Tissue Kit (Invitrogen). 50 ng of DNA was used for each PCR reaction, including 1X PCR buffer containing 1.5 mM MgCl_2_, 0.5 µM of each primer, 1 unit of HotStar Taq polymerase (Qiagen) 0.5 mM dNTP’s, and 2.5 mM of extra MgCl_2_ Primers were: *Nf1^Dsk9^* (forward 5′-GCCAGTAGAAATATCAATGGAAAA-3′, reverse 5′-GGGTGGGGAATCACATACAG-3′, followed by digestion with AflIII) and *Ednrb^s-l^* (S-L forward 5′-CCCTACCCTTCTCACCCACT-3′, S-L reverse 5′- GCATTACCTCAGGCTCCAC-3′, WT forward 5′-CATTTGTCCCAGGGATAGGA-3′, and WT reverse 5′-CAGCTTTTGCTAATGGCTGA-3′).

### Dermal-epidermal Separation and Pixel Intensity of Tail Skin

1 cm piece of skin from the middle of the tail was removed from the tail bones of 2 week old mice, incubated in 2 M sodium bromide for 2 hours at 37°C, and separated using fine forceps. Skin samples to be compared to one another were photographed within a single picture. Dermal skin pigmentation was quantified using ImageJ in terms of average pixel intensity.

### Uveal Melanoma

An archival, paraffin embedded, enucleated uveal melanoma from a patient with neurofibromatosis type 1 was obtained under the approval of the institutional review board at University of British Columbia and the Vancouver General Hospital (previously described in [30].) The data was analyzed anonymously. Tumor tissue from five 8 µM thick sections was micro dissected, digested with proteinase K, and heat inactivated. 1 uL (∼5 ng of DNA) was used to amplify *GNAQ* and *GNA11* exons 4 and 5 as previously described [24], [25].

### Sequencing

5 µL of diluted PCR product (35 ng) was mixed with 2 µL of Exo-SAP-IT PCR product clean-up mix (Affymetrix). 3 µL of Exo-SAPed PCR product, 1 pM of primer, and 3 µL of Big dye mix (v3.1, Applied Biosystems) were used for each sequencing reaction, and run on an Applied Biosystems 3730 DNA Analyzer.

### Statistics

Jump was used to determine that all data was distributed normally using the Shapiro-Wilk W test. p values of statistical significance were calculated using either student’s ttest.
